# Monocyte dysregulation: consequences for hepatic infections

**DOI:** 10.1007/s00281-021-00852-1

**Published:** 2021-04-07

**Authors:** Julie Sellau, Tobias Puengel, Stefan Hoenow, Marie Groneberg, Frank Tacke, Hannelore Lotter

**Affiliations:** 1 Department of Molecular Biology and Immunology, Bernhard Nocht Institute for Tropical Medicine, Hamburg, Germany; 2 Department of Hepatology and Gastroenterology, Charité-Universitätsmedizin Berlin, Campus Virchow-Klinikum and Campus Charité Mitte, Berlin, Germany

**Keywords:** Monocytes, Infection, Inflammation, Hepatic disorder

## Abstract

Liver disorders due to infections are a substantial health concern in underdeveloped and industrialized countries. This includes not only hepatotropic viruses (e.g., hepatitis B, hepatitis C) but also bacterial and parasitic infections such as amebiasis, leishmaniasis, schistosomiasis, or echinococcosis. Recent studies of the immune mechanisms underlying liver disease show that monocytes play an essential role in determining patient outcomes. Monocytes are derived from the mononuclear phagocyte lineage in the bone marrow and are present in nearly all tissues of the body; these cells function as part of the early innate immune response that reacts to challenge by external pathogens. Due to their special ability to develop into tissue macrophages and dendritic cells and to change from an inflammatory to an anti-inflammatory phenotype, monocytes play a pivotal role in infectious and non-infectious liver diseases: they can maintain inflammation and support resolution of inflammation. Therefore, tight regulation of monocyte recruitment and termination of monocyte-driven immune responses in the liver is prerequisite to appropriate healing of organ damage. In this review, we discuss monocyte-dependent immune mechanisms underlying hepatic infectious disorders. Better understanding of these immune mechanisms may lead to development of new interventions to treat acute liver disease and prevent progression to organ failure.

## Background

Due to its high degree of vascularization, the liver functions as a blood-cleansing “sieve,” which is exposed constantly to foreign or self-antigens such as pathogens, toxins, and cell debris; therefore, the organ plays an initial role in balancing and limiting inflammatory responses [1, 2]. To facilitate this, the liver contains large numbers of transient or permanently resident, highly specialized, and effective innate immune cells [3]. First and foremost are intra-sinusoidal macrophages called Kupffer cells (KCs), which have a high capacity for phagocytosis and recognize self or foreign antigens, such as pathogen-associated molecular patterns (PAMPs) or damage-associated molecular patterns (DAMPs), released by necrotic cells [4]. KCs capture circulating bacteria from the bloodstream, as demonstrated for their ability to clear *Staphylococcus aureus* bacteremia [5]. Activation of KCs leads to release of chemokines and to activation as well as recruitment of lymphoid- or myeloid-derived innate immune cells such as natural killer cells, natural killer T cells, neutrophils, and monocytes [3, 6–8]. In contrast to other effector cells, monocytes, which are derived from hematological progenitors in the bone marrow, move to the site of injury or infection via the blood stream and exert multiple functions. For example, these cells can give rise to self-renewing, tissue-resident KCs, which were previously assumed to develop exclusively from embryonic precursor cells [9], as well as tissue-specific macrophages and dendritic cells (DCs) [10]. In addition, these cells are critical for defense against microbial infections and for promoting resolution of inflammation; however, while exerting these actions, they can cause profound collateral damage to host tissues by perpetuating inflammation and promoting fibrosis [8, 11–13].

Accordingly, monocytes represent a unique cell population capable of exceptional plasticity since they can act both as precursors of KCs and as effector cells; they can even switch from one state to another by undergoing marked phenotypic and functional changes [14, 15]. Monocytes and monocyte-derived macrophages are characterized by two major functions. On the one hand, they eliminate pathogens by phagocytosis, release active compounds such as reactive oxygen species, secrete pro-inflammatory cytokines, and modulate T-cell-mediated immune response via antigen presentation [16]. On the other, they are key players in tissue and wound healing reactions because they initiate, maintain, and resolve tissue repair processes [4]. In mice, monocytes are divided into two major subsets: classical (which exhibit pro-inflammatory and antimicrobial properties) and non-classical (which exhibit anti-inflammatory properties and mediate reparative and antiviral mechanisms) [17].

Monocytes are differentiated according to expression of specific surface markers and receptors, which differ between the most investigated species, mice and humans. In mice, classical monocytes express CD11b, high levels of Ly6C, and the CC chemokine receptor 2 (CCR2), but low levels of CX_3_C chemokine receptor 1 (CX_3_CR1) [18]. But even though CX_3_CR1 expression was commonly used as additional marker for classical and non-classical monocyte discrimination in mice, a recent publication demonstrated that both cell types exhibit equal CX_3_CR1 protein expression level on the surface [19]. This discrepancy was referred to the use of mice which express EGFP in monocytes a.o. under control of the endogenous CX_3_CR1 locus [20]. EGFP-positive cells should be considered cells that expressed CX_3_CR1 within the half-life of EGFP (>24 h). This implies that current surface expression of CX_3_CR1 is not correlated proportionally with EGFP fluorescence and the historically common discrimination of murine classical and non-classical monocytes with the help of CX_3_CR1 surface expression should reconsidered [19]. Classical monocytes, which represent approximately 2–5% of circulating white blood cells, are referred to as Ly6C^hi^ monocytes. The non-classical subset of circulating monocytes in mice is slightly less abundant; this subset expresses low levels of Ly6C and high levels of CD43 [12, 19]. In humans, monocyte subsets are divided into three groups according to expression of CD14, the co-receptor for toll-like receptor (TLR) 4 (which recognizes bacterial lipopolysaccharides) [21], and CD16 (also known as FcγRIII) [22]. In addition to CCR2, the human equivalent of classical monocytes is classified as CD14^++^ and CD16^-^, and non-classical monocytes are defined as CD14^+^CD16^++^ and do express higher level of CX3CR1 compared to classical human monocytes [23]. The third subset in humans is a CD14^++^CD16^+^ intermediate population with pro-inflammatory properties [23]. Studies show that there seems to be an intermediate (Ly6C^mid/low^) monocyte subpopulation in mice as well. Detailed genomic characterization revealed that C/EBPß drives conversion of Ly6C^hi^ monocytes to Ly6C^lo^ monocytes via Ly6C^int^ monocytes, which are more heterogenous than the already known populations [15]. Furthermore, studies in a model of sterile hepatic injury in mice which express EGFP under control of the endogenous CX_3_CR1 locus and RFP under the control of CCR2 locus suggest that CCR2^hi^ classical monocytes transition into CX_3_CR1^hi^ monocytes and that this conversion is essential for the role of non-classical Ly6C^lo^ monocytes in ensuring proper tissue repair [24].

Generally, it is accepted that the function of the different monocyte subsets is conserved across species. However, comparison of gene expression profiles between mouse and human monocyte subsets by microarray analysis revealed differences in expression profiles [25]. The use of advanced methods such as fate mapping and single-cell RNA sequencing to decipher population dynamics and functional differences between monocytes suggests that, beyond the historical classification into classical, intermediate, or non-classical subsets, many more subsets might exist [16]. Most recently, a study in a murine model of multiple sclerosis (experimental autoimmune encephalomyelitis) identified at least eight different monocyte subsets by single-cell RNA sequencing [26].

Egress and tissue recruitment of classical monocytes is highly dependent on the surface receptor CCR2, which binds to C-C chemokine ligand 2 (CCL2; also known as MCP1) or CCL7 (also known as MCP3) [11, 27]. Many liver cells are capable of expressing CCL2 and, to a lesser extent, CCL7, and high expression levels are observed in the circulation during infectious or sterile inflammatory conditions [27–29]. Despite different approaches, the exact roles played by CCL2 and CCL7 in recruitment of monocytes are unclear; however, we do know that lack of expression leads to a significant reduction in monocyte numbers during infection [12, 27, 28].

**Fig. 1:**
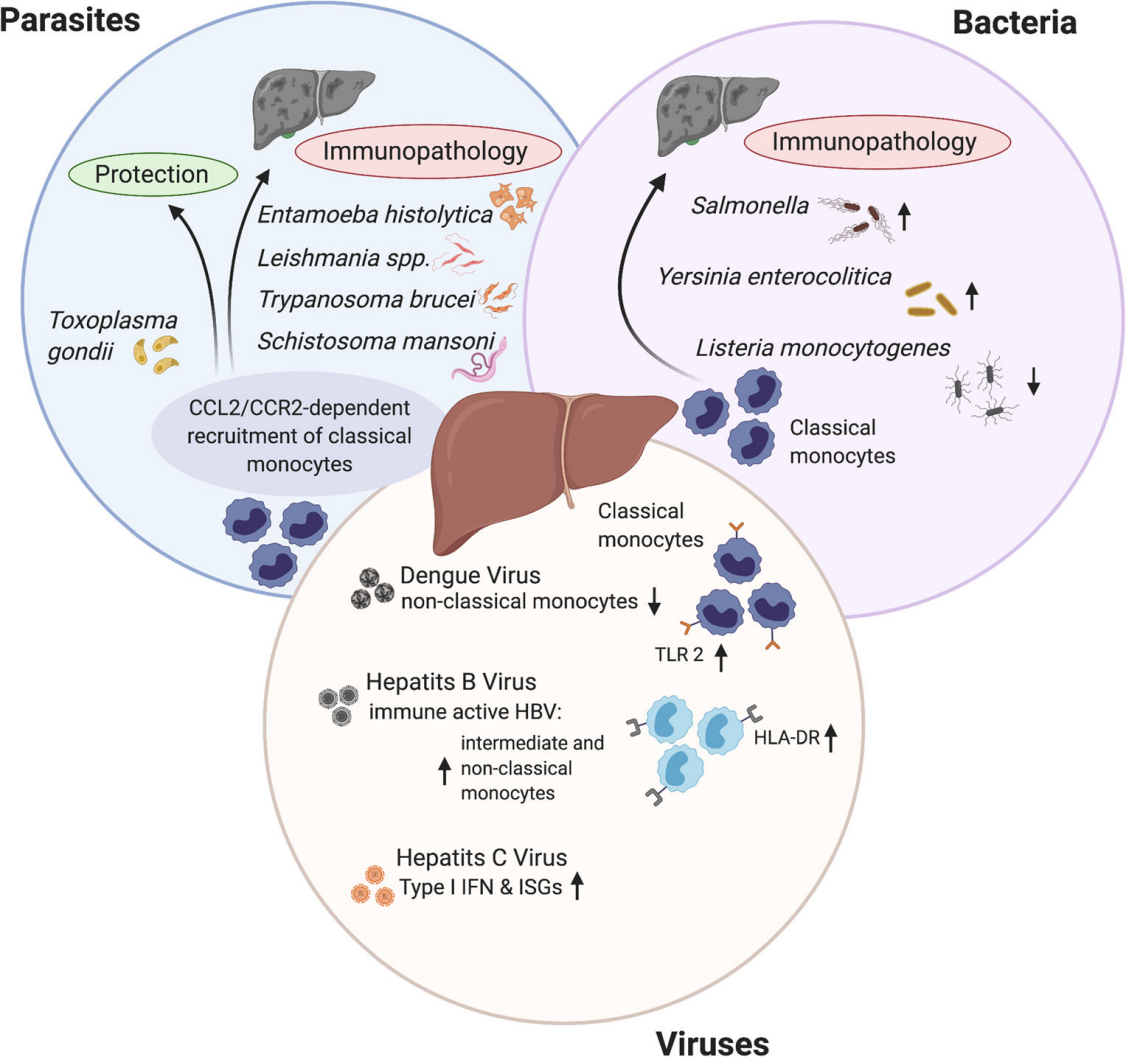
The pivotal role of monocytes in liver infections. Liver infections by pathogens such as parasites, bacteria, and viruses can lead to the recruitment of classical, pro-inflammatory monocytes via the CCL2/CCR2 axis. An important role of pro-inflammatory monocytes is to combat these invading pathogens. However, dysregulation in monocyte recruitment and activation can also lead to the initiation of immunopathologic processes that promote liver injury and favor the survival of pathogens. The figure was created with a licensed version of Biorender.com

Generation of mice harboring a deletion in the *ccr2* gene (*Ccr2*^-/-^ mice) allowed to study the actual role of monocytes in different disease settings [30]. *Ccr2*^-/-^ mice harbor reduced numbers of classical circulating blood monocytes and show impaired monocyte recruitment to various tissues during inflammation; this is concomitant with an increased monocyte population in the bone marrow [27, 30]. The latter finding appears to be due to a defect in adhesion to the vascular endothelium, which inhibits extravasation from the bone marrow [31]. Using this mouse model, the pivotal role of monocytes in different diseases was depicted clearly. On the one hand, they were essential for control and clearance of microbial infections and for resolving inflammatory conditions; however, they also contributed to the pathogenesis of certain infections, drug-induced toxicity, and chronic inflammatory and degenerative diseases [12, 32]. Monocyte-mediated protective and anti-inflammatory responses are seen in many cases of bacterial, parasitic, and viral infections, including listeriosis, tuberculosis, toxoplasmosis, cutaneous leishmaniasis*,* and malaria [33–37], as well as in cases of herpes simplex and West Nile virus infection [38]. However, more and more infection-related diseases have been identified in which monocytes contribute to immunopathology; examples include murine models of protozoan infections such as trypanosomiasis and hepatic amebiasis [39, 40], and human viral infections such as influenza, SARS-CoV, or SARS-CoV2 [41–43]. The ability of monocytes to induce tissue damage results from their primary role in defense against invading pathogens, for which they are equipped with a number of strong effector mechanisms; these include secretion of matrix metalloproteinases capable of degrading extracellular matrix components [44], a high capacity for phagocytosis and antigen presentation [10], release of nitric oxide (NO) [33], reactive oxygen species (ROS) [45], pro-inflammatory cytokines (tumor necrosis factor α (TNF), interleukin (IL)-6, IL-8, and IL-1β), chemokines (CCL1, CCL2, CCL3, and CCL5), and secretion of growth factors (granulocyte colony-stimulating factor and granulocyte-macrophage colony-stimulating factor) [46, 47]. In particular, production of chemokines such as CXC motif ligand 1 (CXCL1; also known as GRO-α oncogene or neutrophil-activating protein 3) can lead to further recruitment of innate immune cells, which maintain the inflammatory process [48]. However, monocytes do not act independently; rather, they are closely associated with activation and polarization of resident macrophages in damaged tissues [49]. Also, as for monocytes, the traditional categorization of macrophages as pro-inflammatory (M1) or anti-inflammatory/tissue repairing (M2) is called into question by new methodologies; a more comprehensive phenotypical description is needed to describe macrophages in different disease settings [49, 50]. For instance, the environment of a diet-induced fatty liver imprints a unique inflammatory signature on various myeloid hepatic cell populations that has features of M1 as well as M2 polarization [51]. Nevertheless, liver macrophages are within the first line of defense against foreign or self-derived challenges, and as such they respond flexibly to signals arising from circulating blood and immune cells [52]. Furthermore, their potential to secrete an armada of immune cell-activating cytokines and chemokines puts them in a position to recruit circulating monocytes to control inflammation, repair tissues, and terminate the inflammatory response, thereby controlling the different stages of liver injury [53]. When the homeostasis of this hepatic system is disturbed, profound immunopathological states, which may lead to severe liver dysfunction, cirrhosis, and fibrosis (which require highly demanding treatment and development of new treatment strategies), can occur [46, 54, 55]. Below, we describe the immunopathological role of monocytes during development of liver-specific infections by different pathogens, including parasites, bacteria, and viruses (Fig. 1). The information provided will consolidate our knowledge of monocyte-induced immunopathologies.

## Infectious microbial challenges affecting the liver

Infectious agents such as protozoan/parasites, bacteria, and viruses can have various effects on the liver. Clinical manifestations range from asymptomatic, characterized by transient increases in aminotransaminases, to massive liver destruction, fibrosis, and development of primary liver tumors [56]. Immunological mechanisms underlying many infection-related diseases of the liver are, for the most part, incompletely understood. For this reason, we review a selection of infection-related liver diseases and focus on those for which involvement of monocytes in development of liver damage has been investigated.

### Monocyte dysfunction during parasitic infections

There are two main classes of parasite: unicellular protozoa and multicellular helminths. Both can cause serious disease in humans during different stages of development. Among the most important protozoa that have the potential to damage the liver are vector-borne pathogens such as *Leishmania* or trypanosomes, and intestinal parasites such as the protozoan *Entamoeba (E.) histolytica*. The second group includes large helminths such as tapeworms, known as cestodes (e.g., *Echinococcus multilocularis*), or flatworms such as *Schistosoma* (e.g., *Schistosoma mansoni*), both of which can cause life-threatening liver disease in humans [57]. Dysregulated and overwhelming inflammation is a major cause of pathogenicity during parasitic infections. Although many studies highlight the role of monocytes in host defense against parasites, only a few have investigated the contribution of monocytes to parasite-mediated liver damage. This is due, at least in part, to the lack of suitable animal models for complex organisms such as parasites, which are highly adapted to their host.

#### Hepatic amebiasis

Invasive amebiasis is caused by oral infection with the environmental-resistant cyst stage of the protozoan parasite *E. histolytica*, which is still a serious health problem in developing countries with poor sanitary conditions; it is also a concern for returning travelers and immigrants [58]. Initially, the intestine is asymptomatically colonized by motile trophozoites, which develop from environment-resistant cysts [59]. Virulence factors expressed by trophozoites, including surface lectins, cysteine peptidases, and pore-forming peptides, enable the parasite to penetrate the mucous membrane wall and induce hemorrhagic diarrhea; the parasite then spreads via the bloodstream to other organs, especially the liver [60]. Liver manifestation is characterized by progressive destruction of liver tissue, resulting in amebic liver abscess, which occurs predominantly in adult men [61]. Several rodent models mimic the liver lesions observed in humans, which are characterized by a central necrotic zone surrounded by a trophozoite layer and a ring of inflammatory cells that delimit the abscess from the liver tissue [62]; however, most studies are hampered by a lack of tools suitable for studying the immunological mechanisms underlying abscess development. An immunocompetent murine model of the disease exhibits high similarity to hepatic amebiasis in humans; however, in contrast to humans, mice are able to control abscess development [63]. Primary histological and immunological studies using this model reveal massive infiltration by immune cells, mainly monocytes and neutrophils, and an increase in expression of CCL2 mRNA and protein in the liver at early time points post-intrahepatic infection with axenically cultured trophozoites [40, 63]. CCL2 expression and monocyte recruitment in this infection model is dependent on initiation of the IL-23/IL-17 axis [64]. Although this axis is involved in protection against several pathogens [65, 66], infection-induced pathological changes can occur after activation [67]. Depletion of CD11b^+^ Ly6C^+^ monocytes and neutrophils from *wild type* mice using Gr1- and Ly6G-specific monoclonal antibodies significantly reduced abscess formation, and liver damage was virtually eliminated [40]. Depletion of liver-resident macrophages by clodronate-loaded liposomes abrogated abscess formation, and depletion of TNF (which is also secreted by Ly6C^hi^ monocytes) revealed that this cytokine is mainly responsible for tissue damage [40]. In addition to TNF, expression of CCL2 and the chemoattractant CXCL1 by Ly6C^hi^ monocytes increased during *E. histolytica* infection [48]. Furthermore, surface molecules expressed by *E. histolytica* might contribute to activation of monocytes and macrophages. Previous *in vitro* studies show that the Gal/GalNAc lectin (Gal-lectin) activates the NF-kB and MAP kinase-signaling pathways in murine macrophages, resulting in upregulation of mRNAs encoding cytokine and receptor genes involved in pro-inflammatory responses [68, 69]. The interaction between another highly immunogenic surface molecule and pathogenicity factor expressed by *E. histolytica* (a lipopeptidophosphoglycan) [70, 71] and TLR2 and TLR4 led to release of IL-10, IL12p40, TNF, and IL-8 by human monocytes, again via activation of NF-kB [47]. IL-8, also known as CXC motif ligand 8 (CXCL8), is a member of the CXC chemokine family that binds to target cells expressing the CXCR1 or CXCR2 receptors, thereby inducing chemotaxis of innate immune cells such as neutrophils and monocytes to sites of infection or injury [72], also contributing to angiogenesis and liver damage [73]. Very closely related to the functions of CXCL8 are those of CXCL1 [74]. Binding of CXCL1 to its specific receptor CXCR2 leads to recruitment of neutrophilic granulocytes and monocytes into inflamed or injured tissues [75, 76]; it also promotes inflammatory processes via regulation of the NLRP3 inflammasome [77]. In *E. histolytica* liver infection, depletion of CXCL1 reduces tissue damage, which is paralleled by diminished recruitment of Ly6C^hi^ monocytes [48].

Moreover, TNF stimulates expression of NADPH oxidase via activation of NF-kB pathways in monocytic cells. Activation of NADP(H) oxidase can lead to generation of superoxide anions, which form oxidants such as hydroxyl radicals (OH•) or hydrogen peroxide (H_2_O_2_) in combination with water [78]. Together with the production of nitric oxide by macrophages, these effectors are highly toxic to amebic trophozoites *in vitro* [79]. However, the parasite is equipped with specific enzymes that allow detoxification of oxygen and nitrogen intermediates [80], and it even expresses an arginase that competes with inducible NO synthase for its substrate [81]. Given these abilities of *E. histolytica* to respond to monocyte/macrophage-mediated defense mechanisms, it seems possible that immune effector molecules may react against host tissues rather than against the parasite. Indeed, depletion of TNF using a specific monoclonal antibody reduces abscess size in the mouse model of amebiasis [40].

Interestingly, expression of TNF and CXCL1 was higher in monocytes from male individuals than in those from females; this applied to both Ly6C^hi^ monocytes in the murine model of amebiasis and CXCL1 in LPS-stimulated CD14^++^CD16^-^ monocytes from healthy human subjects [48]. Moreover, expression of TNF, CCL2, and CXCL1 by Ly6C^hi^ monocytes in the murine model for hepatic amebiasis is increased by testosterone treatment, and expression of TNF and CXCL1 by LPS-stimulated PBMCs from women undergoing testosterone treatment increases while they undergo gender transition [48]. The effect of sex is also observed for CCL2. Serum levels of CCL2 in men intestinally infected with *E. histolytica* are significantly higher than those in women [29]. In addition, transcriptomic data from stimulated human monocytes from both sexes reveals a stronger expression profile for genes associated with monocyte migration, chemotaxis, and motility in men [48].

Repair of liver damage after parasite infection has been investigated in the murine model for hepatic amebiasis; data suggest that repair can be attributed to Ly6C^lo^ monocytes, thereby paralleling observations from repair processes after sterile liver fibrosis [82]. A rapid and significant increase in regenerative IL13- and Arg1-releasing Ly6C^lo^ monocytes upon infection was identified as being involved in abscess recovery [64].

Overall, the data suggest that hepatic amebiasis, which occurs predominately in men, is largely dependent on recruitment of monocytes and monocyte/macrophage-mediated immune activation triggered by testosterone.

#### Leishmaniasis

Leishmaniasis is a vector-borne, wide-spread disease caused by intracellular infection with a protozoan parasite; this disease has three main variants: cutaneous (CL), mucocutaneous (MCL), and visceral leishmaniasis (VL). The latter is a severe systemic parasitosis induced by *Leishmania (L.) donovani* and *L. infantum* [83]. *Leishmania* parasites survive within phagocytic cells such as neutrophils, monocytes, and macrophages and then spread to the spleen, liver, and bone marrow [84]. In the liver, *L. donovani* preferentially infects KCs and macrophages derived from circulating inflammatory monocytes [85]. Protective immunity against VL is mediated by a strong Th1 immune response, characterized by activation of the IL12-IFNγ-inducible (i)NOS pathway triggered by dendritic cells [86]. Interestingly, mice lacking STAT1, an important transcription factor for Th1 signal transduction, are resistant to infection with *L. donovani*, suggesting that additional immune factors are involved in protection [87]. Later on, it was shown that adoptive transfer of wild-type Ly6C^hi^ monocytes to *L. donovani*-infected *STAT1*^-/-^ mice restores the original phenotype of C57BL/6 WT mice [85]. Blocking recruitment of inflammatory monocytes using the CCR2 antagonist RS-504393 leads to a significant reduction in the parasite load in the liver. This was paralleled by a reduction in IFN-γ/IL10-producing CD4^+^ T cells [85]. This immunopathology could be counteracted by IL-17 production of γδ T cells, which are recruited to the liver during *L. donovani* infection and exert a suppressive effect on CCR2^+^ monocytes [88]. Not only in VL but also in CL caused by *L. braziliensis*, the increased frequency of intermediate CD14^++^CD16^+^ monocytes producing TNF and IL-1ß is associated with tissue damage and immunopathology in humans [89, 90]; interestingly, as in amebiasis, infection rates and disease severity caused by the above-mentioned *Leishmania* species are more prominent in men [91].

#### Trypanosomiasis

African trypanosomes are extracellular protozoan parasites that cause sleeping sickness in humans and Nagana disease in cattle in sub-Saharan Africa. Experimental murine models are used to study infection-associated liver pathology of the causative protozoans, *Trypanosoma (T.) brucei* and *T. congolensis,* respectively [92]. In both models, protection requires IFNγ-dependent activation of classically activated monocytic cells and production of trypanotoxic effector molecules such as TNF and/or NO [92]. Overproduction of these effectors by persistent infection leads to liver cell necrosis by TNF- and iNOS-producing DCs (Tip-DCs). These inflammatory Tip-DCs develop either directly from Ly6C^hi^ monocytes or via a maturation step that involves IFNγ and MyD88 signaling [39]. Hence, CCL2/CCR2-dependent recruitment of Ly6C^hi^ monocytes from the bone marrow is a requirement for induction of liver damage, and, accordingly, the absence of CCR2 signaling reduces liver pathogenicity and prolongs survival of *T. brucei*-infected mice [39]. Although CCR5 and macrophage migration inhibitory factor (MIF), two other recruitment factors, were detected on monocytes during murine *T. brucei* infection, it was shown that these factors are not necessary for the emigration of classical monocytes from the bone marrow but promote their migration to the liver [39]. Especially, MIF increases recruitment of inflammatory monocytes and neutrophils and mediates pathogenic inflammatory immune responses during murine *T. brucei* infection [93]. Interestingly, IL-10 plays a major role in *T. brucei* infection. The depletion of IL-10, either using *Il10*^-/-^ mice or through treatment with an antibody specific for the IL-10R, increased expression of CCL2 mRNA and protein, along with the frequency of Ly6C^hi^ monocytes; this led to exacerbation of pathogenicity [39, 94]. Consequently, IL-10 inhibits differentiation of monocytes to Tip-DCs and protects the host from tissue damage [94]. In summary, as in amebiasis and leishmaniasis, the imbalance between host protection and hepatic tissue damage caused by trypanosome infection is mediated by Ly6C^hi^ monocytes.

#### Schistosomiasis

Schistosomiasis is an often neglected tropical disease caused by infection with helminths belonging to the genus *Schistosoma*, especially *Schistosoma (S.) mansoni*, and others [95]. Humans are infected by free-swimming cercaria, which penetrate the skin and migrate to the liver, where young and mature worm stages provoke unspecific reactive hepatitis. Eggs, which are released in the liver parenchyma, trigger an immune response that results in chronic granulomas and periportal fibrosis [96]. These granulomas are characterized by dense accumulation of innate immune cells and cells belonging to the adaptive arm of the immune response.

The murine infection model for the disease is characterized by CCL2/CCR2-dependent recruitment of Ly6C^hi^ monocytes to the liver, and an increase in granuloma-associated resident CX_3_CR1^+^ macrophages. Pulse-chase BrdU labeling revealed that most of the macrophages in the infected liver arise from recruited Ly6C^hi^ monocytes; also, deletion of monocytes (using CCR2-DTR mice) resulted in reduced granuloma formation but exacerbated disease pathogenicity [97].

Peripheral monocytes, which are recruited to the site of infection, produce TGF-β and differentiate into monocyte-derived CX_3_CR1^+^ macrophages, which become alternatively activated due to IL-4/IL-13 signaling via IL-4Rα. This type of macrophage is further characterized by transcription of *Arg1, Chi3l3, Relma,* and *Mrc1*, which is coordinated by STAT6 [98]. Furthermore, these macrophages are potent producers of pro-fibrinogenic cytokines such as IL-10 and TGF-ß. Interestingly, it seems that recruited Ly6C^hi^ monocytes during schistosomiasis transition directly into alternatively activated macrophages without first adopting the phenotype of Ly6C^lo^ monocytes [98]. However, Ly6C^lo^ and intermediate Ly6C cells also contribute to pathogenicity; indeed, reduced hepatic accumulation observed in mice with reduced expression of CD18 (ß_2_ integrin receptor subunit) results in increased formation of granulomatous lesions in the liver, increased egg deposition, and increased mortality [99].

Studies in human patients with incipient or severe fibrosis show high expression of HLA-DR, TGF-ß, IL-6, and TNF, and reduced expression of anti-fibrotic IL-12, by CD14^++^CD16^-^ and CD14^++^CD16^+^ monocytes [96]. This suggests that, analogous to mice, classical monocyte subsets contribute to the pool of alternatively activated macrophages, and hence to liver fibrosis. Indeed, monocyte therapy reduces liver fibrosis induced by *S*. *mansoni* infection in experimental settings, mainly due to a decrease in production of inflammatory and pro-fibrogenic mediators. In addition, monocyte infusion caused downregulation of factors associated with pro-inflammatory macrophages, as well as upregulation of markers associated with alternatively-activated monocytes. These findings reinforce the hypothesis that a predominance of alternatively activated macrophages may favor improvement of hepatic fibrosis in a preclinical model through fibrous tissue remodeling and modulation of the inflammatory response and fibrogenesis [100].

Given that monocytes from males show a stronger pro-inflammatory profile than those from females due to higher testosterone levels [48], and that testosterone is protective in a murine model of schistosomiasis [101], it can be assumed that monocytes play a central role in the immunopathology of *S. mansoni* infection in a sex-dependent manner.

#### Echinococcosis

Infections caused by the cestode *Echinococcus (E.) granulosus* lead to development of hydatide cysts in the liver, which can result in chronic cholangitis or liver cirrhosis [57].

Antigen B (EgAgB), an abundant lipoprotein released by the larvae of *E. granulosus*, is a potential ligand for monocyte and macrophage receptors [102]. Ligation of EgAgB inhibits PMA/LPS-mediated secretion of IL-1β and TNF by THP1 cells; it also induces a modest arginase-I response and triggers differentiation towards an alternative activation-like phenotype. This type of signal transmission could limit TLR4-mediated pro-inflammatory cytokine production by extracellular matrix proteins, which occurs during the growth of cysts, leading to immune evasion by the parasite [103]. By contrast, recruited monocytes in mouse models of potentially fatal liver infection by *E. multilocularis* are characterized by high production of TNF and caspase 3; TUNEL staining reveals significant apoptosis, which may suppress the host antiparasitic immune response [104].

#### Toxoplasmosis

The tachyzoite stage of the protozoan *Toxoplasma (T.) gondii* can manifest in the liver as giant-cell hepatitis or non-specific reactive hepatitis, especially in immunocompromised hosts in whom an infection exacerbates chronic latent infections [57]. However, in contrast to the protozoan infections described above, experimental murine studies suggest that recruitment of Ly6C^hi^, but not neutrophils, contributes to protection from toxoplasmosis, and impaired emigration of monocytes results in severe inflammation and loss of parasite control [105]. Protection is mediated by TNF- and iNOS-producing cells, which differentiate from monocytes recruited to the lamina propria in a CCR2-independent manner [35]. Furthermore, recruited monocytes dampen neutrophil-mediated tissue damage by producing prostaglandin E2 and IL-10 [106]. Also, *T. gondii* profilin (TgPRF), a TLR11 ligand, promotes recruitment of Ly6C^hi^CCR2^+^ inflammatory monocytes and even confers resistance to co-infection by bacteria such as *Listeria monocytogenes* [107].

Interestingly, *T. gondii*-induced immunopathology in the gut is mediated by IL-23, but unlike amebiasis, matrix metalloproteinase-2 and IL-22 (not further activation of the IL-23/IL-17 axis or CCL2-dependent recruitment of monocytes) is essential for tissue damage [108].

Taken together, these parasitic diseases are accompanied by an unbalanced monocytic immune response. In most cases, monocyte recruitment is induced by the CCL2/CCR2 axis, and immunopathology is often triggered by the release of TNF. Due to the scarcity of primary liver tissue from infected patients, the role of monocytes is mostly elucidated in immunocompetent mouse models, so far. In addition, all of these infections suffer from the fact that they are classified as neglected diseases that occur in low-income populations in developing countries.

### Monocyte dysfunction during bacterial infections

Mononuclear phagocytes, including the tissue-resident KCs in the liver, contribute to antibacterial defense mechanism. For instance, they clear blood-circulating bacteria followed by intracellular bacterial killing; in cases of insufficient bactericidal activity, they can also serve as reservoirs for further bacterial spreading [109, 110]. In general, the Ly6C^hi^ monocytes are less likely to trigger immunopathological events in response to bacterial liver infections than in response to parasitic or viral infections. However, such reactions can occur.

#### Listeria monocytogenes

Infection with *Listeria (L.) monocytogenes*, a gram-positive, intracellular bacterium, leads to recruitment of inflammatory cells to the liver and spleen and to formation of liver microgranuloma. Tip-DCs derived from Ly6C^hi^ monocytes contribute to innate defense against *L. monocytogenes*; hence, infection is worse in mice deficient in CCL2 and CCL7 [11] or in *Ccr2*^-/-^ mice due to an increased bacterial burden. Furthermore, T-cell activation in response to *L. monocytogenes* infection is not hampered in the absence of Tip-DCs [33]. Moreover, hepatic TLR2 signaling promotes release of CCL2 and CXCL1 upon *L. monocytogenes* infection, leading to increased motility of monocytes and macrophages, which results in liver microgranuloma formation [111].

#### Salmonella

The intestine is a major target for *Salmonella* spp.; however, this gram-negative bacteria species can spread to other organs and, occasionally, to the liver. Although liver-specific infection studies in mice are rare, data suggest that the enteric phase of infection is characterized by immunopathology mediated by iNOS-producing inflammatory monocytes. Like other enteropathogenic bacteria, *Salmonella* is equipped with type-three secretion systems (T3SS)-1 and T3SS-2. A recent study shows that these complexes interfere with and promote CCL2/CCR2-dependent recruitment of inflammatory monocytes and tissue damage. Pathology and bacterial growth are abolished in *CCR2*^-/-^ mice, suggesting that monocytes might serve as a niche for *Salmonella*, as observed for *Leishmania* [112].

#### Yersinia enterocolitica

Infections with *Yersinia enterocolitica* are not restricted to the gastrointestinal tract and mesenteric lymph nodes; they also affect the liver. Although monocyte-derived CD11c^+^ DCs are essential for priming a protective CD8 T-cell response to this infection, mice lacking CCR2 show better survival, reduced weight loss, and increased clearance of *Yersinia* from the mesenteric lymph nodes. This correlates with reduced expression of Ly6C^hi^ in the periphery, and by increased numbers of neutrophils in the liver [113]. In this case, monocytes may play a dual role: on the one hand, they are involved in establishing a protective immune response, but they may also contribute to immunopathology. However, further detailed experiments are needed to clarify these conflicting results.

In conclusion, several studies report an imbalanced or dysregulated innate immune response to bacterial infection; however, there is a general lack of information about the detailed role of monocytes as a possible cause of immunopathology during responses to these infections.

### Monocyte dysfunction during viral infections

As with certain protozoan or bacterial pathogens, infection by viruses may also lead to manipulation and utilization of monocytes/macrophages for dissemination, long-term persistence within tissues, and replication. Moreover, there are also specialized viruses that specifically infect parenchymal cells of the liver, including hepatocytes.

#### Dengue virus

The mosquito-transmitted *Dengue virus* (DENV), a positive-stranded RNA virus belonging to the family *Flaviviridae*, has four serotypes that cause up to 96 million cases annually; symptoms range from mild febrile episodes to a severe hemorrhagic course that may be accompanied by acute liver failure [56, 114]. In addition to monocytes and immature monocyte-derived DCs, the virus itself and viral antigens can be detected in hepatocytes and KCs, suggesting that these cells support viral replication and contribute to liver injury [114, 115]. The severe course of DENV infections is characterized by endothelial damage and vascular leakage, which results from PAMP/DAMP-mediated production of IL-1ß and TNF by DCs and monocytes [116]. Different subsets of monocytes play an important role in immunopathology, leading to the severe disease outcomes. Studies in humans reveal that infection with DENV reduces both the number and frequency of non-classical CD14^+^CD16^++^ monocytes, which produce TNF and show a more activated phenotype than cells from healthy controls. Furthermore, these non-classical monocytes show decreased expression of CD16, along with an increased expression of CD64, CD86, CCR2, and CCR5, while CX_3_CR1 is reduced upon DENV infection [117]. A recent study shows that DENV infection increases expression of TLR2 by peripheral monocytes which, together with CD14 and TLR6, promote expression of inflammatory cytokines. As a result, vascular permeability increases, paving the way for the severe progressive form of the disease [116].

#### Hepatitis B/hepatitis C (HBV/HCV)

Infection with hepatitis B virus (HBV) and hepatitis C virus (HCV) are among the most important causes of hepatitis and chronic liver disease worldwide [118, 119]. The disease course of HBV and HCV differ with respect to immune responses, leading to distinct clinical outcomes. Infection with the DNA virus HBV, which belongs to the *Hepadnaviridae* family, can be controlled in humans (either naturally or by use of antivirals), but it persists for a lifetime. HCV is a positive-stranded RNA virus belonging to the *Flaviviridae* family; it is a highly potent inducer of chronic persistent infections, which lead (if untreated) to fibrosis. There are two key points concerning treatment of these viruses: while infection with HBV, but not HCV, can be prevented by vaccination, chronic HCV, but not HBV, infection can be successfully eliminated by treatment with direct-acting antiviral drugs. HCV infection induces release of type I (IFNα/β) and type III (IFNλ) interferons, which increase expression of interferon-stimulated genes (ISGs), leading to an antiviral profile in the liver. By contrast, HBV infection does not induce production of interferons; hence, it results in impaired induction of ISG expression [120]. The outcome of HBV infection is determined by the adaptive immune response, represented by HBV-specific antibody-producing B cells, multi-specific polyclonal CD8^+^ cytotoxic T cells, and activated CD4^+^ T cells [121].

HBV induces inflammatory cytokine production by monocytes via TLR2/MyD88/NF-κB signaling but inhibits their IFN-α/β responsiveness and, consequently, effector functions mediated by ISGs; this suggests that, in contrast to HCV, HBV escapes surveillance by the innate immune system [120, 122]. Investigations into the role of monocytes and their contribution to the fate of viral liver infections are performed mainly in circulating monocytes due to the scarcity of HBV- or HCV-infected primary liver tissues and the lack of fully immunocompetent HBV/HCV murine models. Neither the abundance of different monocyte populations nor the function of monocytes in the peripheral blood of chronic HBV patients are altered, nor is their ability to induce T-cell cytokine production and proliferation after TLR stimulation [123, 124]. However, the frequency of intermediate CD14^++^CD16^+^ and non-classical CD14^+^CD16^++^ monocytes in immune-active patients is high and correlates with increased alanine transaminase (ALT) levels and liver histological activity index (HAI) scores, which may indicate a possible role for these cells in liver inflammation and fibrosis during chronic infection. More specifically, intermediate CD14^++^CD16^+^ and non-classical CD14^+^CD16^++^ monocytes in patients with immune-active hepatitis show increased expression of HLA-DR and inflammatory cytokines and have a greater capacity to induce expansion of the Th17 cell population [125]. Monocyte-derived macrophages and KCs in the liver have the potential to further accelerate the pro-inflammatory process; indeed, monocyte-derived macrophages and KCs interact with soluble factors such as hepatitis B surface antigen (HBsAg), resulting in cell activation and cytokine production, including pro-inflammatory cytokines [126]. Moreover, intrahepatic (pro-inflammatory) CD14^+^HLA-DR^hi^CD206^+^ myeloid cells have been linked to HBV-induced liver inflammation and subsequent fibrosis [127, 128]. However, HBsAg counteracts excessive immune activation by monocytes. Furthermore, *in vitro* analysis reveals that HBsAg suppresses pro-inflammatory cytokine production by LPS-stimulated monocytes [129]. HBV particles impair the allostimulatory capacity of monocyte-derived DCs, thereby reducing their potential to stimulate autologous T cells against a recall antigen and suppressing T helper cell type 1 responses [130]. HBeAg, another soluble virus factor, reduces production of TNF and IL-6 by peripheral mononuclear cells after TLR2-challenge [131].

As mentioned previously, HCV infection follows different mechanisms to escape from the immune system. Besides the activation of ISGs during HCV infection, classical monocytes from healthy donors, cultured with HCV-infected hepatoma cells or HCV alone, expressed pro- as well as anti-inflammatory cytokines and showed characteristic surface marker expression of alternatively activated macrophages, including CD206, which was also detected on monocytes from HCV-infected patients. [132]. Elevated hepatic macrophage numbers are characteristic for an HCV infection, which is associated with high levels of pro- and anti-inflammatory cytokines [133]. This explains the dual responses: hepatitis and apoptosis of infected hepatocytes caused by inflammatory cytokines as well as viral persistence due to deactivation of TLR3-mediated antiviral activities like interferon release or TRAIL expression by HCV [134]. Moreover, monocytes from chronically infected HCV patients showed an increased expression of programmed death (PD)-1 which correlated with an impaired IL-12 secretion [135].

Furthermore, during chronic HCV or HBV infection, Kupffer cells produce immunomodulatory mediators that suppress antiviral T-cell responses, such as IL-10, TGFβ, galectin-9, PD-L1, and PD-L2 [136]. Therefore, HCV infection induces an immune response, which favors a persistent of HCV and chronic course of disease.

Nevertheless, conducting functional *in vivo* studies to investigate early innate immune responses against HBV and HCV are challenging due to a lack of immunocompetent mouse models; therefore, lymphocytic choriomeningitis virus (LCMV), which establishes a persistent liver infection in mice, is used as a comparable murine model [137]. LCMV infection induces massive and early recruitment of inflammatory monocytes to the liver and increases liver-specific expression of mRNA encoding pro-inflammatory cytokines. However, compared with those recruited after conventional LPS challenge, LCMV-recruited monocytes are impaired in terms of their phagocytic capacity, and gene expression levels of TNF, IL-6, IL-10, CCL2, IP-10, and CCL5 are similar to those of KCs, which retain their phagocytic capacity after 24 h. The higher proportion of inflammatory monocytes compared with KCs during the early phase of infection suggests that inflammatory monocytes contribute to the intrahepatic inflammatory response, at least in the LCMV infection model [138]. LCMV also counteracts induction of neutralizing humoral immunity. Ly6C^hi^ monocytes interact with LCMV-specific B cells in draining lymph nodes, thereby suppressing antiviral responses and reducing survival via production of NO [139]. During LCMV infection, the ability of inflammatory monocyte-primed CD8 T cells to efficiently produce inflammatory cytokines and granzyme B is reduced, suggesting that monocytes hamper the effector function of CD8 T cells [140].

In summary, several studies have shown that monocytes are affected by viral hepatitis and have a potential role in supporting immunopathology in the liver, especially in HBV-infected individuals; however, at the same time, viral infection may suppress or counteract monocyte activation. Further studies are needed to elucidate the mechanisms that affect the balance between monocyte suppression and activation in viral hepatitis and to identify new intervention points of treatment.

## Conclusion

Adequate and regulated immune reactions are necessary to fight infections and to prevent immunopathology. Hepatic infections are particularly challenging because the liver needs to retain its immunotolerance profile with respect to harmless (but abundant) antigens such as nutrients from the intestinal system. Monocytes are part of innate immune response against pathogens that cause hepatic diseases. Recent findings show that rather than being categorized into only three groups, monocytes should be described as a population of highly diverse cells that have strong adaptive potential and exceptional plasticity with respect to combating injury and disease. Nevertheless, for many diseases, the CCR2/CCL2 axis plays a crucial role in recruiting monocytes to the site of injury. The described hepatic infections have one thing in common: an imbalance in the responses of monocytes, which has a crucial impact on disease course. As some infectious agents such as *Leishmania* parasites or DENV target monocytes directly, the diseases activate monocytes, which target invading parasites or infected cells via three major mechanisms: phagocytosis, antigen presentation to T cells, and cytokine expression. However, insufficient control of monocyte activation leads to immunopathology, as described for amebiasis and leishmaniasis, whereas suppression of monocytes triggers development of fibrosis (as in schistosomiasis and hepatitis B and C).

Directly targeting the function of monocytes to reduce immunopathology and cure diseases remains challenging [136]; however, a better understanding of the interaction between monocytes and the complex hepatic system is expected to identify new points of intervention and new treatments for hepatic diseases.

## References

[1] Heymann F, Tacke F (2016). Immunology in the liver--from homeostasis to disease. Nat Rev Gastroenterol Hepatol.

[2] Strnad P, Tacke F, Koch A, Trautwein C (2017). Liver - guardian, modifier and target of sepsis. Nat Rev Gastroenterol Hepatol.

[3] Kubes P, Jenne C (2018). Immune responses in the liver. Annu Rev Immunol.

[4] Tacke F, Zimmermann HW (2014). Macrophage heterogeneity in liver injury and fibrosis. J Hepatol.

[5] Zeng Z, Surewaard BG, Wong CH, Geoghegan JA, Jenne CN, Kubes P (2016). CRIg Functions as a macrophage pattern recognition receptor to directly bind and capture blood-borne gram-positive bacteria. Cell Host Microbe.

[6] Jenne CN, Kubes P (2013). Immune surveillance by the liver. Nat Immunol.

[7] Su L, Li N, Tang H, Lou Z, Chong X, Zhang C, Su J, Dong X (2018). Kupffer cell-derived TNF-alpha promotes hepatocytes to produce CXCL1 and mobilize neutrophils in response to necrotic cells. Cell Death Dis.

[8] Brempelis KJ, Crispe IN (2016). Infiltrating monocytes in liver injury and repair. Clin Transl Immunol.

[9] Scott CL, Zheng F, De Baetselier P, Martens L, Saeys Y, De Prijck S, Lippens S, Abels C, Schoonooghe S, Raes G, Devoogdt N, Lambrecht BN, Beschin A, Guilliams M (2016). Bone marrow-derived monocytes give rise to self-renewing and fully differentiated Kupffer cells. Nat Commun.

[10] Jakubzick CV, Randolph GJ, Henson PM (2017). Monocyte differentiation and antigen-presenting functions. Nat Rev Immunol.

[11] Serbina NV, Pamer EG (2006). Monocyte emigration from bone marrow during bacterial infection requires signals mediated by chemokine receptor CCR2. Nat Immunol.

[12] Shi C, Pamer EG (2011). Monocyte recruitment during infection and inflammation. Nat Rev Immunol.

[13] Karlmark KR, Tacke F, Dunay IR (2012). Monocytes in health and disease - Minireview. Eur J Microbiol Immunol (Bp).

[14] Wynn TA, Vannella KM (2016). Macrophages in tissue repair, regeneration, and fibrosis. Immunity.

[15] Mildner A, Schonheit J, Giladi A, David E, Lara-Astiaso D, Lorenzo-Vivas E, Paul F, Chappell-Maor L, Priller J, Leutz A, Amit I, Jung S (2017). Genomic characterization of murine monocytes reveals C/EBPbeta transcription factor dependence of Ly6C(-) cells. Immunity.

[16] Guilliams M, Mildner A, Yona S (2018). Developmental and functional heterogeneity of monocytes. Immunity.

[17] Auffray C, Sieweke MH, Geissmann F (2009). Blood monocytes: development, heterogeneity, and relationship with dendritic cells. Annu Rev Immunol.

[18] Geissmann F, Jung S, Littman DR (2003). Blood monocytes consist of two principal subsets with distinct migratory properties. Immunity.

[19] Meghraoui-Kheddar A, Barthelemy S, Boissonnas A, Combadière C (2020). Revising CX3CR1 expression on murine classical and non-classical monocytes. Front Immunol.

[20] Jung S, Aliberti J, Graemmel P, Sunshine MJ, Kreutzberg GW, Sher A, Littman DR (2000). Analysis of fractalkine receptor CX3CR1 function by targeted deletion and green fluorescent protein reporter gene insertion. Mol Cell Biol.

[21] Zanoni I, Granucci F (2013). Role of CD14 in host protection against infections and in metabolism regulation. Front Cell Infect Microbiol.

[22] Janeway CA, Medzhitov R (2002). Innate immune recognition. Annu Rev Immunol.

[23] Ziegler-Heitbrock L, Ancuta P, Crowe S, Dalod M, Grau V, Hart DN, Leenen PJ, Liu YJ, MacPherson G, Randolph GJ, Scherberich J, Schmitz J, Shortman K, Sozzani S, Strobl H, Zembala M, Austyn JM, Lutz MB (2010). Nomenclature of monocytes and dendritic cells in blood. Blood.

[24] Dal-Secco D, Wang J, Zeng Z, Kolaczkowska E, Wong CH, Petri B, Ransohoff RM, Charo IF, Jenne CN, Kubes P (2015). A dynamic spectrum of monocytes arising from the in situ reprogramming of CCR2+ monocytes at a site of sterile injury. J Exp Med.

[25] Ingersoll MA, Spanbroek R, Lottaz C, Gautier EL, Frankenberger M, Hoffmann R, Lang R, Haniffa M, Collin M, Tacke F, Habenicht AJ, Ziegler-Heitbrock L, Randolph GJ (2010). Comparison of gene expression profiles between human and mouse monocyte subsets. Blood.

[26] Giladi A, Wagner LK, Li H, Dorr D, Medaglia C, Paul F, Shemer A, Jung S, Yona S, Mack M, Leutz A, Amit I, Mildner A (2020). Cxcl10(+) monocytes define a pathogenic subset in the central nervous system during autoimmune neuroinflammation. Nat Immunol.

[27] Tsou CL, Peters W, Si Y, Slaymaker S, Aslanian AM, Weisberg SP, Mack M, Charo IF (2007). Critical roles for CCR2 and MCP-3 in monocyte mobilization from bone marrow and recruitment to inflammatory sites. J Clin Invest.

[28] Jia T, Serbina NV, Brandl K, Zhong MX, Leiner IM, Charo IF, Pamer EG (2008). Additive roles for MCP-1 and MCP-3 in CCR2-mediated recruitment of inflammatory monocytes during Listeria monocytogenes infection. J Immunol.

[29] Bernin H, Marggraff C, Jacobs T, Brattig N, Le VA, Blessmann J, Lotter H (2014). Immune markers characteristic for asymptomatically infected and diseased Entamoeba histolytica individuals and their relation to sex. BMC Infect Dis.

[30] Boring L, Gosling J, Chensue SW, Kunkel SL, Farese RV, Broxmeyer HE, Charo IF (1997). Impaired monocyte migration and reduced type 1 (Th1) cytokine responses in C-C chemokine receptor 2 knockout mice. J Clin Invest.

[31] Kuziel WA, Morgan SJ, Dawson TC, Griffin S, Smithies O, Ley K, Maeda N (1997). Severe reduction in leukocyte adhesion and monocyte extravasation in mice deficient in CC chemokine receptor 2. Proc Natl Acad Sci U S A.

[32] Zimmermann H, Trautwein C, Tacke F (2012) Functional role of monocytes and macrophages for the inflammatory response in acute liver injury. Front Physiol 3(56) 10.3389/fphys.2012.00056 PMC3475871 23091461

[33] Serbina NV, Salazar-Mather TP, Biron CA, Kuziel WA, Pamer EG (2003). TNF/iNOS-producing dendritic cells mediate innate immune defense against bacterial infection. Immunity.

[34] Peters W, Scott HM, Chambers HF, Flynn JL, Charo IF, Ernst JD (2001). Chemokine receptor 2 serves an early and essential role in resistance to Mycobacterium tuberculosis. Proc Natl Acad Sci U S A.

[35] Dunay IR, Damatta RA, Fux B, Presti R, Greco S, Colonna M, Sibley LD (2008). Gr1(+) inflammatory monocytes are required for mucosal resistance to the pathogen Toxoplasma gondii. Immunity.

[36] Conrad SM, Strauss-Ayali D, Field AE, Mack M, Mosser DM (2007). Leishmania-derived murine monocyte chemoattractant protein 1 enhances the recruitment of a restrictive population of CC chemokine receptor 2-positive macrophages. Infect Immun.

[37] Sponaas AM, do Rosario APF, Voisine C, Mastelic B, Thompson J, Koernig S, Jarra W, Renia L, Mauduit M, Potocnik AJ, Langhorne J (2009). Migrating monocytes recruited to the spleen play an important role in control of blood stage malaria. Blood.

[38] Terry RL, Getts DR, Deffrasnes C, van Vreden C, Campbell IL, King NJ (2012). Inflammatory monocytes and the pathogenesis of viral encephalitis. J Neuroinflammation.

[39] Bosschaerts T, Guilliams M, Stijlemans B, Morias Y, Engel D, Tacke F, Hérin M, De Baetselier P, Beschin A (2010). Tip-DC development during parasitic infection is regulated by IL-10 and requires CCL2/CCR2, IFN-gamma and MyD88 signaling. PLoS Pathog.

[40] Helk E, Bernin H, Ernst T, Ittrich H, Jacobs T, Heeren J, Tacke F, Tannich E, Lotter H (2013). TNFalpha-mediated liver destruction by Kupffer cells and Ly6Chi monocytes during Entamoeba histolytica infection. PLoS Pathog.

[41] Coates BM, Staricha KL, Koch CM, Cheng Y, Shumaker DK, Budinger GRS, Perlman H, Misharin AV, Ridge KM (2018). Inflammatory monocytes drive influenza a virus-mediated lung injury in juvenile mice. J Immunol.

[42] Channappanavar R, Fett C, Mack M, Ten Eyck PP, Meyerholz DK, Perlman S (2017). Sex-based differences in susceptibility to severe acute respiratory syndrome coronavirus infection. J Immunol.

[43] Merad M, Martin JC (2020). Pathological inflammation in patients with COVID-19: a key role for monocytes and macrophages. Nat Rev Immunol.

[44] Newby AC (2008). Metalloproteinase expression in monocytes and macrophages and its relationship to atherosclerotic plaque instability. Arterioscler Thromb Vasc Biol.

[45] Watanabe T, Yasunari K, Nakamura M, Maeda K (2006). Carotid artery intima-media thickness and reactive oxygen species formation by monocytes in hypertensive patients. J Hum Hypertens.

[46] Triantafyllou E, Woollard KJ, McPhail MJW, Antoniades CG, Possamai LA (2018). The role of monocytes and macrophages in acute and acute-on-chronic liver failure. Front Immunol.

[47] Maldonado-Bernal C, Kirschning CJ, Rosenstein Y, Rocha LM, Rios-Sarabia N, Espinosa-Cantellano M, Becker I, Estrada I, Salazar-González RM, López-Macías C, Wagner H, Sánchez J, Isibasi A (2005). The innate immune response to Entamoeba histolytica lipopeptidophosphoglycan is mediated by toll-like receptors 2 and 4. Parasite Immunol.

[48] Sellau J, Groneberg M, Fehling H, Thye T, Hoenow S, Marggraff C, Weskamm M, Hansen C, Stanelle-Bertram S, Kuehl S, Noll J, Wolf V, Metwally NG, Hagen SH, Dorn C, Wernecke J, Ittrich H, Tannich E, Jacobs T, Bruchhaus I, Altfeld M, Lotter H (2020). Androgens predispose males to monocyte-mediated immunopathology by inducing the expression of leukocyte recruitment factor CXCL1. Nat Commun.

[49] Murray PJ, Allen JE, Biswas SK, Fisher EA, Gilroy DW, Goerdt S, Gordon S, Hamilton JA, Ivashkiv LB, Lawrence T, Locati M, Mantovani A, Martinez FO, Mege JL, Mosser DM, Natoli G, Saeij JP, Schultze JL, Shirey KA, Sica A, Suttles J, Udalova I, van Ginderachter JA, Vogel SN, Wynn TA (2014). Macrophage activation and polarization: nomenclature and experimental guidelines. Immunity.

[50] Xue J, Schmidt SV, Sander J, Draffehn A, Krebs W, Quester I, De Nardo D, Gohel TD, Emde M, Schmidleithner L, Ganesan H, Nino-Castro A, Mallmann MR, Labzin L, Theis H, Kraut M, Beyer M, Latz E, Freeman TC, Ulas T, Schultze JL (2014). Transcriptome-based network analysis reveals a spectrum model of human macrophage activation. Immunity.

[51] Krenkel O, Hundertmark J, Abdallah AT, Kohlhepp M, Puengel T, Roth T, Branco DPP, Mossanen JC, Luedde T, Trautwein C, Costa IG, Tacke F (2020). Myeloid cells in liver and bone marrow acquire a functionally distinct inflammatory phenotype during obesity-related steatohepatitis. Gut.

[52] Davies LC, Jenkins SJ, Allen JE, Taylor PR (2013). Tissue-resident macrophages. Nat Immunol.

[53] Liaskou E, Wilson DV, Oo YH (2012). Innate immune cells in liver inflammation. Mediat Inflamm.

[54] van der Heide D, Weiskirchen R, Bansal R (2019). Therapeutic targeting of hepatic macrophages for the treatment of liver diseases. Front Immunol.

[55] Krenkel O, Puengel T, Govaere O, Abdallah AT, Mossanen JC, Kohlhepp M, Liepelt A, Lefebvre E, Luedde T, Hellerbrand C, Weiskirchen R, Longerich T, Costa IG, Anstee QM, Trautwein C, Tacke F (2018). Therapeutic inhibition of inflammatory monocyte recruitment reduces steatohepatitis and liver fibrosis. Hepatology.

[56] Talwani R, Gilliam BL, Howell C (2011). Infectious diseases and the liver. Clin Liver Dis.

[57] Hawash Y, Radu-Ionita PNF, Jinga M, Tintoui I, Su Z, Bontas E (2020). Parasitic liver disease. Liver Diseases.

[58] Shirley DT, Farr L, Watanabe K, Moonah S (2018). A review of the global burden, new diagnostics, and current therapeutics for amebiasis. Open Forum Infect Dis.

[59] Zulfiqar H, Mathew G, Horrall S (2020). Amebiasis.

[60] Faust DM, Guillen N (2012). Virulence and virulence factors in Entamoeba histolytica, the agent of human amoebiasis. Microbes Infect.

[61] Blessmann J, Van Linh P, Nu PA, Thi HD, Muller-Myhsok B, Buss H, Tannich E (2002). Epidemiology of amebiasis in a region of high incidence of amebic liver abscess in central Vietnam. Am J Trop Med Hyg.

[62] Ventura-Juarez J, Jarillo-Luna RA, Fuentes-Aguilar E, Pineda-Vazquez A, Munoz-Fernandez L, Madrid-Reyes JI, Campos-Rodriguez R (2003). Human amoebic hepatic abscess: in situ interactions between trophozoites, macrophages, neutrophils and T cells. Parasite Immunol.

[63] Lotter H, Jacobs T, Gaworski I, Tannich E (2006). Sexual dimorphism in the control of amebic liver abscess in a mouse model of disease. Infect Immun.

[64] Noll J, Helk E, Fehling H, Bernin H, Marggraff C, Jacobs T, Huber S, Pelczar P, Ernst T, Ittrich H, Otto B, Mittrucker HW, Holscher C, Tacke F, Bruchhaus I, Tannich E, Lotter H (2016). IL-23 prevents IL-13-dependent tissue repair associated with Ly6C(lo) monocytes in Entamoeba histolytica-induced liver damage. J Hepatol.

[65] Khader SA, Pearl JE, Sakamoto K, Gilmartin L, Bell GK, Jelley-Gibbs DM, Ghilardi N, deSauvage F, Cooper AM (2005). IL-23 compensates for the absence of IL-12p70 and is essential for the IL-17 response during tuberculosis but is dispensable for protection and antigen-specific IFN-gamma responses if IL-12p70 is available. J Immunol.

[66] Indramohan M, Sieve AN, Break TJ, Berg RE (2012). Inflammatory monocyte recruitment is regulated by interleukin-23 during systemic bacterial infection. Infect Immun.

[67] Kotloski NJ, Nardelli DT, Peterson SH, Torrealba JR, Warner TF, Callister SM, Schell RF (2008). Interleukin-23 is required for development of arthritis in mice vaccinated and challenged with Borrelia species. Clin Vaccine Immunol.

[68] Kammanadiminti SJ, Mann BJ, Dutil L, Chadee K (2004). Regulation of Toll-like receptor-2 expression by the Gal-lectin of Entamoeba histolytica. FASEB J.

[69] Seguin R, Mann BJ, Keller K, Chadee K (1995). Identification of the galactose-adherence lectin epitopes of Entamoeba histolytica that stimulate tumor necrosis factor-alpha production by macrophages. Proc Natl Acad Sci U S A.

[70] Isibasi A, Blanco F, Arreguin C, Martinez G, Pelayo R, Orozco E, Kumate J (1990). Immunochemical differences in the surface polysaccharides obtained from Entamoeba histolytica strain HM1:IMSS and its virulent (C-A) and non-virulent (L-6) clones. Arch Invest Med (Mex).

[71] Lotter H, Gonzalez-Roldan N, Lindner B, Winau F, Isibasi A, Moreno-Lafont M, Ulmer AJ, Holst O, Tannich E, Jacobs T (2009). Natural killer T cells activated by a lipopeptidophosphoglycan from Entamoeba histolytica are critically important to control amebic liver abscess. PLoS Pathog.

[72] Van Sweringen HL, Sakai N, Tevar AD, Burns JM, Edwards MJ, Lentsch AB (2011). CXC chemokine signaling in the liver: impact on repair and regeneration. Hepatology.

[73] Lai WK, Adams DH (2005). Angiogenesis and chronic inflammation; the potential for novel therapeutic approaches in chronic liver disease. J Hepatol.

[74] Vries MH, Wagenaar A, Verbruggen SE, Molin DG, Dijkgraaf I, Hackeng TH, Post MJ (2015). CXCL1 promotes arteriogenesis through enhanced monocyte recruitment into the peri-collateral space. Angiogenesis.

[75] Ritzman AM, Hughes-Hanks JM, Blaho VA, Wax LE, Mitchell WJ, Brown CR (2010). The chemokine receptor CXCR2 ligand KC (CXCL1) mediates neutrophil recruitment and is critical for development of experimental Lyme arthritis and carditis. Infect Immun.

[76] Wang L, Zhang YL, Lin QY, Liu Y, Guan XM, Ma XL, Cao HJ, Liu Y, Bai J, Xia YL, Du J, Li HH (2018). CXCL1-CXCR2 axis mediates angiotensin II-induced cardiac hypertrophy and remodelling through regulation of monocyte infiltration. Eur Heart J.

[77] Boro M, Balaji KN (2017). CXCL1 and CXCL2 regulate NLRP3 inflammasome activation via G-protein-coupled receptor CXCR2. J Immunol.

[78] Gauss KA, Nelson-Overton LK, Siemsen DW, Gao Y, DeLeo FR, Quinn MT (2007). Role of NF-kappaB in transcriptional regulation of the phagocyte NADPH oxidase by tumor necrosis factor-alpha. J Leukoc Biol.

[79] Seguin R, Mann BJ, Keller K, Chadee K (1997) The tumor necrosis factor alpha-stimulating region of galactose-inhibitable lectin of Entamoeba histolytica activates gamma interferon-primed macrophages for amebicidal activity mediated by nitric oxide. Infect Immun 65(7):2522–2527 10.1128/iai.65.7.2522-2527.1997 PMC175356 9199414

[80] Bruchhaus I, Richter S, Tannich E (1998). Recombinant expression and biochemical characterization of an NADPH:flavin oxidoreductase from Entamoeba histolytica. Biochem J.

[81] Elnekave K, Siman-Tov R, Ankri S (2003). Consumption of L-arginine mediated by Entamoeba histolytica L-arginase (EhArg) inhibits amoebicidal activity and nitric oxide production by activated macrophages. Parasite Immunol.

[82] Ramachandran P, Pellicoro A, Vernon MA, Boulter L, Aucott RL, Ali A, Hartland SN, Snowdon VK, Cappon A, Gordon-Walker TT, Williams MJ, Dunbar DR, Manning JR, van Rooijen N, Fallowfield JA, Forbes SJ, Iredale JP (2012). Differential Ly-6C expression identifies the recruited macrophage phenotype, which orchestrates the regression of murine liver fibrosis. Proc Natl Acad Sci U S A.

[83] Dos Santos Meira C, Gedamu L (2019) Protective or detrimental? Understanding the role of host immunity in leishmaniasis. Microorganisms 7(12) 10.3390/microorganisms7120695 PMC6956275 31847221

[84] Gupta G, Oghumu S, Satoskar AR (2013). Mechanisms of immune evasion in leishmaniasis. Adv Appl Microbiol.

[85] Terrazas C, Varikuti S, Oghumu S, Steinkamp HM, Ardic N, Kimble J, Nakhasi H, Satoskar AR (2017). Ly6C(hi) inflammatory monocytes promote susceptibility to Leishmania donovani infection. Sci Rep.

[86] Liu D, Uzonna JE (2012). The early interaction of Leishmania with macrophages and dendritic cells and its influence on the host immune response. Front Cell Infect Microbiol.

[87] Rosas LE, Snider HM, Barbi J, Satoskar AA, Lugo-Villarino G, Keiser T, Papenfuss T, Durbin JE, Radzioch D, Glimcher LH, Satoskar AR (2006). Cutting edge: STAT1 and T-bet play distinct roles in determining outcome of visceral leishmaniasis caused by Leishmania donovani. J Immunol.

[88] Sheel M, Beattie L, Frame TC, de Labastida Rivera F, Faleiro RJ, Bunn PT, Montes de Oca M, Edwards CL, Ng SS, Kumar R, Amante FH, Best SE, McColl SR, Varelias A, Kuns RD, MacDonald KP, Smyth MJ, Haque A, Hill GR, Engwerda CR (2015). IL-17A-producing gammadelta T cells suppress early control of parasite growth by monocytes in the liver. J Immunol.

[89] Passos S, Carvalho LP, Costa RS, Campos TM, Novais FO, Magalhaes A, Machado PR, Beiting D, Mosser D, Carvalho EM, Scott P (2015). Intermediate monocytes contribute to pathologic immune response in Leishmania braziliensis infections. J Infect Dis.

[90] Santos D, Campos TM, Saldanha M, Oliveira SC, Nascimento M, Zamboni DS, Machado PR, Arruda S, Scott P, Carvalho EM, Carvalho LP (2018). IL-1beta production by intermediate monocytes is associated with immunopathology in cutaneous leishmaniasis. J Invest Dermatol.

[91] Lockard RD, Wilson ME, Rodriguez NE (2019). Sex-related differences in immune response and symptomatic manifestations to infection with Leishmania species. J Immunol Res.

[92] Bosschaerts T, Guilliams M, Stijlemans B, De Baetselier P, Beschin A (2009). Understanding the role of monocytic cells in liver inflammation using parasite infection as a model. Immunobiology.

[93] Stijlemans B, Leng L, Brys L, Sparkes A, Vansintjan L, Caljon G, Raes G, Van Den Abbeele J, Van Ginderachter JA, Beschin A, Bucala R, De Baetselier P (2014). MIF contributes to Trypanosoma brucei associated immunopathogenicity development. PLoS Pathog.

[94] Guilliams M, Movahedi K, Bosschaerts T, VandenDriessche T, Chuah MK, Hérin M, Acosta-Sanchez A, Ma L, Moser M, Van Ginderachter JA, Brys L, De Baetselier P, Beschin A (2009). IL-10 dampens TNF/inducible nitric oxide synthase-producing dendritic cell-mediated pathogenicity during parasitic infection. J Immunol.

[95] Colley DG, Bustinduy AL, Secor WE, King CH (2014). Human schistosomiasis. Lancet.

[96] Fernandes JS, Araujo MI, Lopes DM, de Souza Rda P, Carvalho EM, Cardoso LS (2014). Monocyte subsets in schistosomiasis patients with periportal fibrosis. Mediat Inflamm.

[97] Nascimento M, Huang SC, Smith A, Everts B, Lam W, Bassity E, Gautier EL, Randolph GJ, Pearce EJ (2014). Ly6Chi monocyte recruitment is responsible for Th2 associated host-protective macrophage accumulation in liver inflammation due to schistosomiasis. PLoS Pathog.

[98] Souza COS, Gardinassi LG, Rodrigues V, Faccioli LH (2020). Monocyte and macrophage-mediated pathology and protective immunity during schistosomiasis. Front Microbiol.

[99] Souza COS, Espíndola MS, Fontanari C, Prado MKB, Frantz FG, Rodrigues V, Gardinassi LG, Faccioli LH (2018). CD18 Regulates monocyte hematopoiesis and promotes resistance to experimental schistosomiasis. Front Immunol.

[100] de Souza VCA, Moura DMN, de Castro M, Bozza PT, de Almeida Paiva L, Fernandes CJB, Leao RLC, Lucena JP, de Araujo RE, de Melo Silva AJ, Figueiredo R, de Oliveira SA (2019). Adoptive transfer of bone marrow-derived monocytes ameliorates schistosoma mansoni -induced liver fibrosis in mice. Sci Rep.

[101] Nakazawa M, Fantappie MR, Freeman GL, Eloi-Santos S, Olsen NJ, Kovacs WJ, Secor WE, Colley DG (1997). Schistosoma mansoni: susceptibility differences between male and female mice can be mediated by testosterone during early infection. Exp Parasitol.

[102] Díaz A, Casaravilla C, Barrios AA, Ferreira AM (2016). Parasite molecules and host responses in cystic echinococcosis. Parasite Immunol.

[103] Silva-Álvarez V, Folle AM, Ramos AL, Kitano ES, Iwai LK, Corraliza I, Córsico B, Ferreira AM (2016). Echinococcus granulosus antigen B binds to monocytes and macrophages modulating cell response to inflammation. Parasit Vectors.

[104] Yang HQ, Ma SB, Bian ZY, Li J, Zou H, Zhang SJ, Peng XY, Chen XP (2012). Expression of tumor necrosis factor-alpha and caspase-3 protein in monocytes adjacent to the invaded Echinococcus multilocularis in liver. Zhongguo Ji Sheng Chong Xue Yu Ji Sheng Chong Bing Za Zhi.

[105] Dunay IR, Fuchs A, Sibley LD (2010). Inflammatory monocytes but not neutrophils are necessary to control infection with Toxoplasma gondii in mice. Infect Immun.

[106] Grainger JR, Wohlfert EA, Fuss IJ, Bouladoux N, Askenase MH, Legrand F, Koo LY, Brenchley JM, Fraser ID, Belkaid Y (2013). Inflammatory monocytes regulate pathologic responses to commensals during acute gastrointestinal infection. Nat Med.

[107] Neal LM, Knoll LJ (2014). Toxoplasma gondii profilin promotes recruitment of Ly6Chi CCR2+ inflammatory monocytes that can confer resistance to bacterial infection. PLoS Pathog.

[108] Muñoz M, Heimesaat MM, Danker K, Struck D, Lohmann U, Plickert R, Bereswill S, Fischer A, Dunay IR, Wolk K, Loddenkemper C, Krell HW, Libert C, Lund LR, Frey O, Hölscher C, Iwakura Y, Ghilardi N, Ouyang W, Kamradt T, Sabat R, Liesenfeld O (2009). Interleukin (IL)-23 mediates Toxoplasma gondii-induced immunopathology in the gut via matrixmetalloproteinase-2 and IL-22 but independent of IL-17. J Exp Med.

[109] Surewaard BG, Deniset JF, Zemp FJ, Amrein M, Otto M, Conly J, Omri A, Yates RM, Kubes P (2016). Identification and treatment of the Staphylococcus aureus reservoir in vivo. J Exp Med.

[110] Jorch SK, Surewaard BG, Hossain M, Peiseler M, Deppermann C, Deng J, Bogoslowski A, van der Wal F, Omri A, Hickey MJ, Kubes P (2019). Peritoneal GATA6+ macrophages function as a portal for Staphylococcus aureus dissemination. J Clin Invest.

[111] Wang G, Zhao H, Zheng B, Li D, Yuan Y, Han Q, Tian Z, Zhang J (2019) TLR2 promotes monocyte/macrophage recruitment into the liver and microabscess formation to limit the spread of listeria monocytogenes. Front Immunol 10(1388) 10.3389/fimmu.2019.01388 PMC6607897 31297109

[112] McLaughlin PA, Bettke JA, Tam JW, Leeds J, Bliska JB, Butler BP, van der Velden AWM (2019). Inflammatory monocytes provide a niche for Salmonella expansion in the lumen of the inflamed intestine. PLoS Pathog.

[113] Zhang Y, Khairallah C, Sheridan BS, van der Velden AWM, Bliska JB (2018) CCR2(+) Inflammatory monocytes are recruited to Yersinia pseudotuberculosis pyogranulomas and dictate adaptive responses at the expense of innate immunity during oral infection. Infect Immun 86(3) 10.1128/IAI.00782-17 PMC5820931 29263104

[114] Thepparit C, Smith DR (2004). Serotype-specific entry of dengue virus into liver cells: identification of the 37-kilodalton/67-kilodalton high-affinity laminin receptor as a dengue virus serotype 1 receptor. J Virol.

[115] Huerre MR, Lan NT, Marianneau P, Hue NB, Khun H, Hung NT, Khen NT, Drouet MT, Huong VT, Ha DQ, Buisson Y, Deubel V (2001). Liver histopathology and biological correlates in five cases of fatal dengue fever in Vietnamese children. Virchows Arch.

[116] Aguilar-Briseño JA, Upasani V, Ellen BMT, Moser J, Pauzuolis M, Ruiz-Silva M, Heng S, Laurent D, Choeung R, Dussart P, Cantaert T, Smit JM, Rodenhuis-Zybert IA (2020). TLR2 on blood monocytes senses dengue virus infection and its expression correlates with disease pathogenesis. Nat Commun.

[117] Naranjo-Gómez JS, Castillo JA, Rojas M, Restrepo BN, Diaz FJ, Velilla PA, Castaño D (2019). Different phenotypes of non-classical monocytes associated with systemic inflammation, endothelial alteration and hepatic compromise in patients with dengue. Immunology.

[118] Ganem D, Prince AM (2004). Hepatitis B virus infection--natural history and clinical consequences. N Engl J Med.

[119] Thimme R, Oldach D, Chang KM, Steiger C, Ray SC, Chisari FV (2001). Determinants of viral clearance and persistence during acute hepatitis C virus infection. J Exp Med.

[120] Shin EC, Sung PS, Park SH (2016). Immune responses and immunopathology in acute and chronic viral hepatitis. Nat Rev Immunol.

[121] Bertoletti A, Ferrari C (2016). Adaptive immunity in HBV infection. J Hepatol.

[122] Song H, Tan G, Yang Y, Cui A, Li H, Li T, Wu Z, Yang M, Lv G, Chi X, Niu J, Zhu K, Crispe IN, Su L, Tu Z (2019). Hepatitis B virus–induced imbalance of inflammatory and antiviral signaling by differential phosphorylation of STAT1 in human monocytes. J Immunol.

[123] Maini MK, Gehring AJ (2016). The role of innate immunity in the immunopathology and treatment of HBV infection. J Hepatol.

[124] Boltjes A, Groothuismink ZM, van Oord GW, Janssen HL, Woltman AM, Boonstra A (2014). Monocytes from chronic HBV patients react in vitro to HBsAg and TLR by producing cytokines irrespective of stage of disease. PLoS One.

[125] Zhang JY, Zou ZS, Huang A, Zhang Z, Fu JL, Xu XS, Chen LM, Li BS, Wang FS (2011). Hyper-activated pro-inflammatory CD16 monocytes correlate with the severity of liver injury and fibrosis in patients with chronic hepatitis B. PLoS One.

[126] Boltjes A, van Montfoort N, Biesta PJ, Op den Brouw ML, Kwekkeboom J, van der Laan LJ, Janssen HL, Boonstra A, Woltman AM (2015). Kupffer cells interact with hepatitis B surface antigen in vivo and in vitro, leading to proinflammatory cytokine production and natural killer cell function. J Infect Dis.

[127] Tan-Garcia A, Wai LE, Zheng D, Ceccarello E, Jo J, Banu N, Khakpoor A, Chia A, Tham CYL, Tan AT, Hong M, Keng CT, Rivino L, Tan KC, Lee KH, Lim SG, Newell EW, Pavelka N, Chen J, Ginhoux F, Chen Q, Bertoletti A, Dutertre CA (2017). Intrahepatic CD206(+) macrophages contribute to inflammation in advanced viral-related liver disease. J Hepatol.

[128] Tan-Garcia A, Lai F, Sheng Yeong JP, Irac SE, Ng PY, Msallam R, Tatt Lim JC, Wai LE, Tham CYL, Choo SP, Lim T, Young DY, D'Ambrosio R, Degasperi E, Perbellini R, Newell E, Le Bert N, Ginhoux F, Bertoletti A, Chen Q, Dutertre CA (2020). Liver fibrosis and CD206(+) macrophage accumulation are suppressed by anti-GM-CSF therapy. JHEP Rep.

[129] Wang S, Chen Z, Hu C, Qian F, Cheng Y, Wu M, Shi B, Chen J, Hu Y, Yuan Z (2013). Hepatitis B virus surface antigen selectively inhibits TLR2 ligand-induced IL-12 production in monocytes/macrophages by interfering with JNK activation. J Immunol.

[130] Beckebaum S, Cicinnati VR, Zhang X, Ferencik S, Frilling A, Grosse-Wilde H, Broelsch CE, Gerken G (2003). Hepatitis B virus-induced defect of monocyte-derived dendritic cells leads to impaired T helper type 1 response in vitro: mechanisms for viral immune escape. Immunology.

[131] Visvanathan K, Skinner NA, Thompson AJ, Riordan SM, Sozzi V, Edwards R, Rodgers S, Kurtovic J, Chang J, Lewin S, Desmond P, Locarnini S (2007). Regulation of Toll-like receptor-2 expression in chronic hepatitis B by the precore protein. Hepatology.

[132] Saha B, Kodys K, Szabo G (2016). Hepatitis C virus-induced monocyte differentiation into polarized m2 macrophages promotes stellate cell activation via TGF-β. Cell Mol Gastroenterol Hepatol.

[133] McGuinness PH, Painter D, Davies S, McCaughan GW (2000). Increases in intrahepatic CD68 positive cells, MAC387 positive cells, and proinflammatory cytokines (particularly interleukin 18) in chronic hepatitis C infection. Gut.

[134] Tu Z, Pierce RH, Kurtis J, Kuroki Y, Crispe IN, Orloff MS (2010). Hepatitis C virus core protein subverts the antiviral activities of human Kupffer cells. Gastroenterology.

[135] Zhang Y, Ma CJ, Ni L, Zhang CL, Wu XY, Kumaraguru U, Li CF, Moorman JP, Yao ZQ (2011). Cross-talk between programmed death-1 and suppressor of cytokine signaling-1 in inhibition of il-12 production by monocytes/macrophages in hepatitis c virus infection. J Immunol.

[136] Tacke F (2017). Targeting hepatic macrophages to treat liver diseases. J Hepatol.

[137] Zhou X, Ramachandran S, Mann M, Popkin DL (2012). Role of lymphocytic choriomeningitis virus (LCMV) in understanding viral immunology: past, present and future. Viruses.

[138] Movita D, van de Garde MD, Biesta P, Kreefft K, Haagmans B, Zuniga E, Herschke F, De Jonghe S, Janssen HL, Gama L, Boonstra A, Vanwolleghem T (2015). Inflammatory monocytes recruited to the liver within 24 hours after virus-induced inflammation resemble Kupffer cells but are functionally distinct. J Virol.

[139] Sammicheli S, Kuka M, Di Lucia P, de Oya NJ, De Giovanni M, Fioravanti J, Cristofani C, Maganuco CG, Fallet B, Ganzer L, Sironi L, Mainetti M, Ostuni R, Larimore K, Greenberg PD, de la Torre JC, Guidotti LG, Iannacone M (2016) Inflammatory monocytes hinder antiviral B cell responses. Sci Immunol 1(4) 10.1126/sciimmunol.aah6789 PMC5111729 27868108

[140] Shin K-S, Park Y-J, Koh C-H, Bae E-A, Kim I-K, Song B, Seo H, Jeon I, Kang C-Y (2017). IFN-γ-induced MHCII+ inflammatory monocytes play a role for down-regulating CD8 T cell responses in acute LCMV infection. J Immunol.

